# Regulation of Nociceptive Plasticity Threshold and DARPP-32 Phosphorylation in Spinal Dorsal Horn Neurons by Convergent Dopamine and Glutamate Inputs

**DOI:** 10.1371/journal.pone.0162416

**Published:** 2016-09-09

**Authors:** Itsaso Buesa, Zigor Aira, Jon Jatsu Azkue

**Affiliations:** Department of Neurosciences, School of Medicine and Dentistry, University of the Basque Country (UPV/EHU), 48940 Leioa, Bizkaia, Spain; University of Texas at Dallas, UNITED STATES

## Abstract

Dopamine can influence NMDA receptor function and regulate glutamate-triggered long-term changes in synaptic strength in several regions of the CNS. In spinal cord, regulation of the threshold of synaptic plasticity may determine the proneness to undergo sensitization and hyperresponsiveness to noxious input. In the current study, we increased endogenous dopamine levels in the dorsal horn by using re-uptake inhibitor GBR 12935. During the so-induced hyperdopaminergic transmission, conditioning low-frequency (1 Hz) stimulation (LFS) to the sciatic nerve induced long-term potentiation (LTP) of C-fiber-evoked potentials in dorsal horn neurons. The magnitude of LTP was attenuated by blockade of either dopamine D1-like receptors (D1LRs) by with SCH 23390 or NMDA receptor subunit NR2B with antagonist Ro25-6981. Conditioning LFS during GBR 12935 administration increased phosphorylation of dopamine- and cAMP-regulated phosphoprotein of Mr 32kDa (DARPP-32) at threonine 34 residue in synaptosomal (P3) fraction of dorsal horn homogenates, as assessed by Western blot analysis, which was partially prevented by NR2B blockade prior to conditioning stimulation. Conditioning LFS also was followed by higher co-localization of phosphorylated form of NR2B at tyrosine 1472 and pDARPP-32^Thr34^- with postsynaptic marker PSD-95 in transverse L5 dorsal horn sections. Such increase could be significantly attenuated by D1LR blockade with SCH 23390. The current results support that coincidental endogenous recruitment of D1LRs and NR2B in dorsal horn synapses plays a role in regulating afferent-induced nociceptive plasticity. Parallel increases in DARPP-32 phosphorylation upon LTP induction suggests a role for this phosphoprotein as intracellular detector of convergent D1L- and NMDA receptor activation.

## Introduction

Synaptic plasticity at glutamatergic synapses, including long-term potentiation (LTP) of nociceptive neurotransmission, is held as a critical cellular generator of spinal sensitization and persistent pain states [1,2,3,4]. Modification of synaptic plasticity threshold may enable subthreshold conditioning stimuli to effectively trigger LTP, as shown in cortical or hippocampal neurons [5,6,7]. Functional status of the NMDA receptor, intracellular calcium buffering, kinase/phosphatase activities or priming of protein synthesis machinery may operate as mechanisms of regulation of synaptic plasticity [8].

Dopamine can lower the threshold of glutamate-triggered long-term changes in synaptic strength in both hippocampus and amygdala [6,9]. Synaptic actions of dopamine are mediated by five different G-protein coupled receptor subtypes (D1 through 5) that are divided into two major subclasses, viz. the D1-like (D1LRs) and D2-like receptors (D2LRs), which couple to Gs and Gi mediated intracellular signaling systems. At the spinal cord level, dopamine exerts complex actions in the modulation of centripetal transmission of noxious signals. Thus, D2LR activation inhibits afferent input [10,11,12,13], whereas D1LRs is involved in consolidating neural plastic changes triggered by inflammatory pain [14] and regulates opioid receptor-mediated modulation [15]. Recent work has revealed that sustained dopaminergic, D1LR-mediated input to the spinal dorsal horn can shift the NMDA receptor to an enhanced activation state and lower the threshold of synaptic potentiation of C-fiber-evoked excitation and [15,16]. These data imply that increased dopaminergic neurotransmission at the dorsal horn, which has been shown to occur in states of sustained pain [17], holds the potential to profoundly alter nociception by increasing proneness to synaptic plasticity.

Cumulative evidence suggests that dopamine may strongly influence NMDA receptor function in the CNS. Dopamine- and cAMP-regulated phosphoprotein of Mr 32kDa (DARPP-32), a well established target of the synaptic actions of dopamine, is a probable mediator of intracellular cross-talk of dopamine and glutamate signals [18,19]. Dopamine-induced phosphorylation of DARPP-32 can promote its phosphatase-inhibitory activity [20,21]. Particularly, cAMP-dependent PKA signaling pathway triggered by D1R activation phosphorylates the Thr^34^ residue of DARPP-32 [22,23], and this phosporylated form both depletes protein phosphatase-1 (PP1) activity and contributes to maintenance the phosphorylated configuration of NMDA receptor [18,19].

The current study was undertaken to evaluate the role of D1LRs and NMDA receptors in regulating the threshold of synaptic plasticity of C-fiber-evoked excitation in dorsal horn neurons during increased dopaminergic neurotransmission. In addition, we assessed how recruitment of D1LRs and NMDA receptors influence DARPP-32 phosphorylation at residue Thr^34^ and phosphorylation of NMDA receptor subunit 2B at Tyr^1472^ at synaptic compartment in dorsal horn neurons during hyperdopaminergic transmission.

## Materials and Methods

Animal experiments were performed according to the European Communities Council Directive (86/609/ECC) on adult male *Sprague Dawley* rats (250–350 g). The protocols for animal care and use were approved by the appropriate committee at the University of the Basque Country (UPV/EHU).

### Electrophysiology

Procedures were performed under urethane anesthesia (1.5 g/kg, i.p.). A tracheotomy was performed to maintain an open, low-resistance airway, and cannulae were inserted into the left common carotid artery and the right internal jugular vein for arterial blood pressure monitoring (mean 80–100 mmHg) and continuous infusion of Tyrode’s solution (in mM: 137 NaCl, 2.7 KCl, 1.4 CaCl2, 1 MgCl2, 6 NaHCO3, 2.1 NaH2PO4; pH 7.4) at 0.8–1 ml/h, respectively. Colorectal temperature was continuously monitored and euthermia (37–38°C) was maintained via a feedback-controlled underbody heating pad for the duration of the experimental procedure. The left sciatic nerve was exposed, gently freed from connective tissue, and placed onto platinum hook electrodes for bipolar electrical stimulation. Bilateral dorsal laminectomies were performed at vertebrae T13–L1, the vertebral column was immobilized to a rigid frame, and the dura mater overlaying lumbosacral spinal segments was carefully removed.

Electrophysiological setup was essentially as described previously [24]. Tungsten microelectrodes (5 MΩ) were placed into laminae I–II (100–300 μm deep and 1 mm lateral to the spinal mid-line). The position of the tip of the recording electrode in the spinal cord was marked with a small electrolytic lesion by delivery of an anodal current through the recording electrode (50 μA anodal current for 10 s) and histologically verified. Single monophasic, square-wave electrical pulses were delivered as test stimuli to the sciatic nerve trunk at a midthigh level on a per-minute basis by means of a current-controlled stimulus isolator, and the elicited spinal field potentials were amplified (analog bandpass set at 1–550 Hz), displayed on an oscilloscope, and digitized at 10 kS/s and 12-bit resolution (PCI-MIO-16E acquisition card, National Instruments, Austin, TX). Field potentials were evoked in superficial laminae of the spinal dorsal horn by suprathreshold, electrical C-fiber stimulation (3–3.5 mA pulses of 0.5 ms duration) and quantified as described previously [25]. In experiments administering drugs via spinal superfusion (cf. below), each drug concentration change lasted for 20 min, and only the last 10 evoked field potentials were extracted for analysis from the baseline control period and from each treatment period. The areas of field potentials evoked during each treatment period were compared with those recorded during a control, aCSF superfusion period, by using univariant ANOVA and post hoc Bonferroni’s or Tamhane’s multiple-comparison tests. In experiments aimed at inducing LTP of C-fiber-evoked field potentials, drugs were administered starting 30 min prior to conditioning low frequency stimulation, which consisted of two, 30-second trains of 3 mA pulses of 1.5 ms duration at 1 Hz, 30 s apart.

### Drug preparation and delivery

Drugs used included dopamine re-uptake inhibitor GBR 12935 (1-(2-Diphenylmethoxyethyl)-4-(3-phenylpropyl) piperazine dihydrochloride), D1LR antagonist SCH 23390 (7-chloro-3-methyl-1-phenyl-1,2,4,5-tetrahydro-3-benzazepin-8-ol), NR2B antagonist Ro 25–6981 ((αR,βS)-α-(4-Hydroxyphenyl)-β-methyl-4-(phenylmethyl)-1- piperidinepropanol maleate), all from Tocris (Bristol, UK). Stock solutions were obtained by diluting drug powder in double-distilled water, and working solutions were prepared in artificial CSF (aCSF) (in mM: 130 NaCl, 3.5 KCl, 1.25 NaH_2_PO_4_, 24 NaHCO_3_, 1.2 CaCl_2_, 1.2 MgSO_4_, 10 D-(±) glucose; pH 7.4) immediately before delivery. All drug were administered in small volumes (10–15 μl) by controlled superfusion via a silicone, 40–50 mm^2^ pool attached to the dorsal surface of the spinal cord [26]. SCH 23390 and Ro 25–6981 concentrations were selected on the basis of prior published work [16] and preliminary experiments.

### Subcellular fractionation of spinal cord tissue

Biochemical fractionation of dorsal horn proteins was performed with minor variations according to previous studies [27,28]. Briefly, rats were deeply anesthetized with sodium pentobarbital (50 mg/kg, i.p.) and killed by decapitation. L4–L5 segments were quickly extracted into ice-cold aCSF. Tissue was separated and homogenized mechanically with a motor-driven glass/glass tissue homogenizer in ice-cold lysis buffer (10 mM Tris, pH 7.6, 320 mM sucrose, 5 mM EDTA) containing protease inhibitors (5 mM EGTA, 1 mM PMSF, 10 U/ml aprotinin, 0.0001% chymostatin, 0.0001% leupeptin, and 0.0001% pepstatin). Dorsal horn samples ipsilateral and contralateral to surgery were taken and processed separately. Homogenates were centrifuged at 1000 g for 10 min to remove cell nuclei (P1) from the low supernatant (S1). S1 was collected and centrifuged at 10,000 g during 15 min to separate a P2 pellet containing the crude synaptosomal fraction and a cytoplasmic fraction S2 with microsomes. The P2 pellet was incubated in the lysis buffer containing 0.5% Triton and centrifuged at 32,000 g for 20 min to obtain the crude synaptic vesicle fraction (S3) and the final pellet containing the synaptic fraction (P3). The latter was solubilized in resuspension buffer (10 mM Tris, pH 8.0, 1 mM EDTA, 2% SDS). All fractions were stored at 80°C. We have shown previously that only P3 fraction is enriched with synaptic density proteins such as postsynaptic density protein PSD-95 [29].

### Western blot

BCA protein assay kit (Pierce, Rockford, IL) was used for determining protein concentration. Identical amounts of protein (50 μg) were loaded to SDS-PAGE using 8% running gels and transferred to nitrocellulose membranes (GE Healthcare, Barcelona, Spain). After a blocking step with 5% non-fat milk in PBST for 1 h at room temperature, membranes were incubated overnight at 4°C with primary antibody. We used an affinity-purified goat polyclonal antibody against Thr 34 phosphorylated DARPP-32 (sc-21601 from Santa Cruz Biotechnology; Santa Cruz, CA) at 1:1,000 as primary antibody. After incubation with primary antibodies, membranes were washed three times in PBST for 10 min and incubated with HRP-conjugated donkey anti-goat antisera (GE Healthcare, Barcelona, Spain) 1:5,000 for 1 h at room temperature. Thermo Fisher Scientific SuperSignal Chemiluminescent Substrate was used to detect HRP on the blots. Spectrophotometry was used to determine protein concentration in each sample, and the required volume was then calculated to load the same amount of protein (50 μg) to each lane. Reversible validated Ponceau staining was used to check equal loading of gels [30,31]. For quantitation, protein band densities were analyzed by using *NIH ImageJ* software. Student’s *t*-test was used for comparisons.

### Immunofluorescence

Deeply anesthetized rats (sodium pentobarbital; 50 mg/kg, i.p.) were perfused transcardially with 250 ml of 0.9% saline followed by 900 ml of 4% paraformaldehyde in phosphate buffer (PB) (0.1 M), pH 7.4. L4–L5 segments were removed, postfixed with 4% paraformaldehyde in PB for 4 h, and then cryoprotected for 48 h with 30% sucrose in PBS at 4°C. Coronal, 40-μm-thick cryotome sections were serially collected in PBS and preincubated with 1% bovine serum albumin (Sigma, St. Louis, MO) and 1% normal serum (1 h at RT). Three primary antibodies were used in triple immunoflurescece experiments, namely goat polyclonal to Thr 34 phosphorylated DARPP-32 (sc-21601 from Santa Cruz Biotechnology; Santa Cruz, CA) at 1:1.000, mouse monoclonal to PSD-95 (MAB1596 from Millipore, Bedford, MA) at 1:500, and a polyclonal antibody raised in rabbit against NR2B subunit phosphorylated at Tyr1472 (454583 from Millipore). After preincubations with normal serum of species other than those in which the secondary antibodies were raised, sections were sequentially incubated with Cy5 650-conjugated donkey anti-rabbit, Dylight 549-conjugated donkey anti-mouse, and Alexa 488-conjugated donkey anti-goat fluorescent antibodies (1:200; Jackson ImmunoResearch, West Grove, PA) and mounted in Mowiol (Vector Laboratories, Burlingame, CA). Immunolabeled sections were viewed in a Fluoview FV500 Olympus confocal microscope, and digital photomicrographs were acquired sequentially to avoid overlapping of fluorescent emission spectra. *NIH ImageJ* software (*Intensity Correlation* plug-in) [32] was used to adjust brightness and contrast, to obtain image co-localization overlays, as well as to perform pixel-wise intensity correlation-based analyses of confocal photomicrographs. Pearson’s correlation coefficient and Fisher’s exact test were used to determine and contrast co-localization values, respectively.

## Results

### Blockade of either NR2B or D1LRs attenuates LFS-induced LTP during hyperdopaminergic transmission

**Fig 1:**
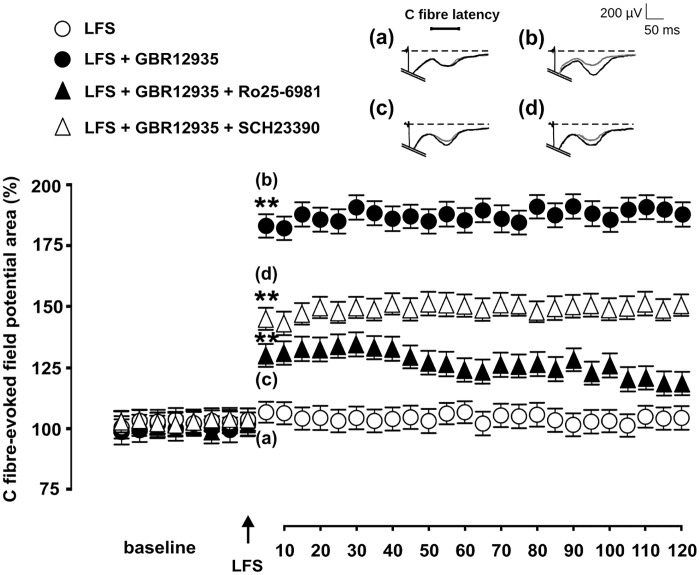
Blockade of either D1LRs or NR2B attenuates LFS-induced LTP of C-fiber-evoked potentials during GBR 12935-induced hyperdopaminergic transmission. Mean areas of C-fiber-evoked potentials are shown prior and after conditioning LFS during spinal superfusion with dopamine re-uptake inhibitor GBR 12935 (10 μM) either alone (solid circles) or in combination with D1LR antagonist SCH 23390 (at 1 μM, open triangles) or NR2B antagonist Ro25-6981 (at 100 μM, solid triangles). Asterisks indicate statistical significance at p<0.01 using the post hoc Bonferroni test following one-way ANOVA, relative to baseline prior to conditioning LFS (n = 6 each group; error bars indicate SEM). Representative recordings obtained 10 min after conditioning stimulation in the four experimental conditions are shown at the top (scale 50 ms, 200 mV; horizontal bar delimits C-fiber latency).

Conditioning low-frequency (1 Hz) electrical stimulation to the sciatic nerve consistently failed to alter the magnitude of evoked potentials in naive rats. In contrast, this same conditioning stimulation protocol induced LTP of C-fiber-evoked potentials during hyperdopaminergic transmission induced by spinal superfusion with dopamine re-uptake inhibitor GBR 12935 at 10 μM (80.20 ± 0.48% increase; p < 0.01 relative to baseline). Administration of GBR 12935 alone in separate control experiments did not significantly alter C-fiber-evoked potentials (15.43 ±0.49 μV· ms potential area during superfusion with 10 μM GBR 12935 vs 15.61±0.38 μV· ms area during superfusion with aCSF). Whenever 1 μM SCH 23390 had been added to the superfusate, LFS induced significant (p<0.01 relative to baseline) yet milder potentiation of evoked responses (45.98% ± 0.32% increase from baseline, p<0.01 relative to LTP induced in the absence of SCH 23390; Fig 1).

To test for the involvement of NR2B in LFS-induced LTP during hyperdopaminergic transmission, we administered 100 μM Ro25-6981 in combination with GBR 12935. We found that LFS still induced significant potentiation of C-fiber-evoked potentials (28.94 ± 0.33% increase from baseline; p <0.01 versus baseline), however synaptic potentiation was significantly lesser than during superfusion with GBR 12935 alone (Fig 1). Neither SCH 23390 or Ro25-6981 affected evoked potentials when administered alone at the concentrations used here, as ascertained in separate control experiments.

### LFS-induced increase in pDARPP-32 in dorsal horn synapses during hyperdopaminergic transmission is partially prevented by NR2B blockade

**Fig 2:**
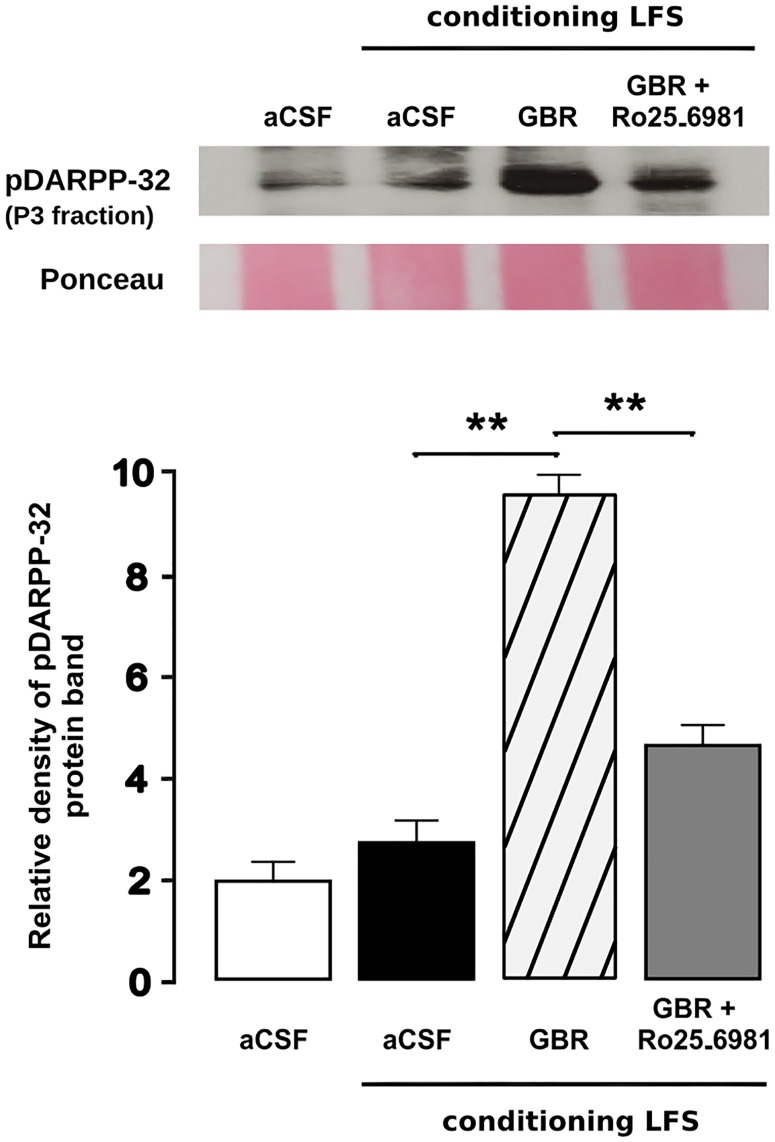
NR2B blockade reduces LFS-induced DARPP-32 phosphorylation at threonine 34 in dorsal horn synapses. Western blot of synaptosomal (P3) fraction of dorsal horn homogenates reveal higher pDARPP-32^Thr34^-like immunoreactivity in rats receiving conditioning LFS during spinal superfusion with GBR 12935 relative to unconditioned rats or to those receiving conditioning stimulation during superfusion with aCSF. Increased immunoreactivity for pDARPP-32^Thr34^ in the former was attenuated whenever Ro25-6981 had been added to the spinal superfusate prior to LFS. Image analysis of immunostained band densities confirmed significantly higher immunoreactivity (asterisks indicate significant difference at *p*<0.01 using the Student's *t*-test) in rats receiving conditioning LFS during spinal superfusion with GBR 12935.

We carried out Western blots on dorsal horn homogenates extracted 120 min after application of conditioning LFS in rats subjected to superfusion with either GBR 12935 for 30 min or aCSF, in order to evaluate DARPP-32 phosphorylation at residue Thr^34^. We found that conditioning stimulation did not alter immunoreactivity for pDARPP-32 in synaptosomal fraction dorsal horn homogenates from control rats previously superfused with aCSF. In contrast, a dramatic increase in pDARPP-32-like immunoreactivity was found in homogenates from rats subjected to GBR 12935 superfusion (Fig 2). As assessed by density analysis, such increase was statistically significant relative to aCSF-superfused rats (p < 0.01 using the Student´s *t*-test; Fig 2). To determine whether NR2B activation was required for increasing DARPP-32 phosphorylation, we used GBR 12935 and Ro25-6981 to simultaneously increase dopamine levels and block NR2B prior to and during conditioning LFS. By using band density analysis we found that immunoreactivity for pDARPP-32 was significantly attenuated relative to rats superfused only with GBR 12935 (p < 0.01 using Student's *t*-test; Fig 2).

### LFS-induced enrichment in pNR2B at pDARPP32-containing postsynaptic sites is dependent on recruitment of D1LRs

Prior evidence indicates that intracellular signaling linked to D1LRs promotes NR2B phosphorylation in prefrontal cortex neurons [33,34] and that NR2B phosphorylation is pivotal in glutamate-dependent central sensitization [35,36]. We assessed whether administration of LFS during hyperdopaminergic transmission could induce NR2B phosphorylation at residue Tyr1472 at synaptic densities containing pDARPP-32. We found that LFS increased NR2B phosphorylation at postsynaptic densities marked by PSD-95 in rats pretreated with GBR 12935 but not in untreated rats (Rr value of 0.78 with GBR 12935 pretreatment relative to 0.04 in absence of GBR 12935, z = -116.21, p< 0.01 at the Fisher's exact test).

**Fig 3:**
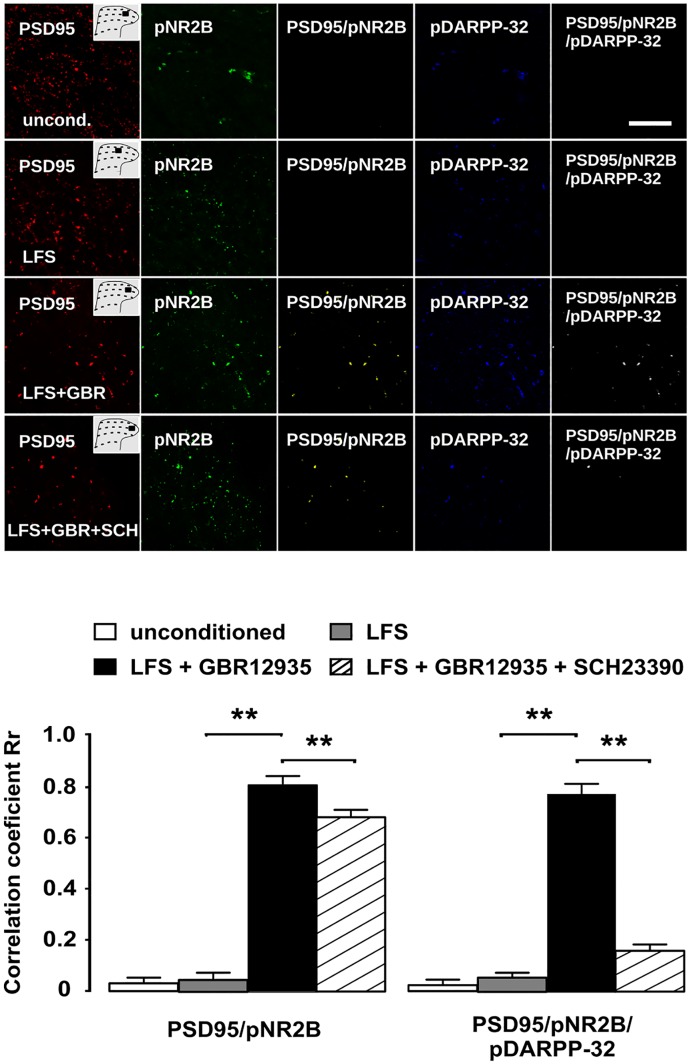
D1LR blockade prevents LFS-induced NR2B phosphorylation on Tyr1472 at postsynaptic, pDARPP-32^Thr34^-containing sites. High-power micrographs (scale bar equals 5 μm) of the superficial L5 dorsal horn from rats either unconditioned or receiving conditioning LFS during spinal superfusion with GBR 12935 alone or in combination with SCH 23390, showing triple immunolabeling for pDARPP-32^Thr34^ (blue), pNR2B^Tyr1472^, (green) and postsynaptic marker PSD-95 (red). Co-localization of pNR2B^Tyr1472^- and PSD-95 immunoreactivities is displayed as yellow overlay, whereas the white overlay indicates triple co-localization with pDARPP-32^Thr34^. Solid boxes in the dorsal horn drawings indicate the sites from which micrographs were taken. Intensity correlation analysis revealed significantly higher PSD-95/pNR2B^Tyr1472^ and PSD-95/pNR2B^Tyr1472^/pDARPP-32^Thr34^ co-localizations in rats receiving conditioning LFS during GBR 12935-induced hyperdopaminergic transmission (p < 0.01 using the Fisher’s exact test for comparison of Pearson’s correlation coefficients). Both increases were significantly attenuated (p < 0.01, Fisher’s exact test) if D1LRs had been blocked by co-administering SCH 23390 prior to conditioning LFS. Error bars indicate SEM.

As shown in Fig 3, pDARPP-32 and pNR2B were hardly co-localized with postsynaptic marker PSD-95 in dorsal horn sections from either conditioned or unconditioned, otherwise untreated rats. However, PSD-95/pNR2B/pDARPP32 co-localization was found to be increased dramatically in tissue from rats that had received conditioning LFS during hyperdopaminergic transmission induced by spinal superfusion with GBR 12935 (Rr 0.73 vs 0.04 in those receiving LFS alone; z = -111.42, p < 0.01 using the Fisher's exact test). Concurrent blockade of D1LRs, achieved by adding SCH 23390 to the spinal superfusate prior to conditioning LFS, resulted in a lower increase in PSD-95/pNR2B/pDARPP32 co-localization (Rr 0.17 vs 0.73 in the absence of SCH 23390, z =, p<0.01).

## Discussion

In the present work, we investigated how synaptic activation of D1LRs and NMDA receptor subunit 2B can contribute to regulating the threshold of afferent-induced synaptic plasticity in dorsal horn neurons. In addition, we evaluated parallel changes in DARPP-32 phosphorylation at threonine 34 and its co-localization with the phosphorylated form of NR2B at tyrosine 1472.

### Concurrent activation of D1LRs and NR2B decreases the threshold of afferent-induced synaptic plasticity in dorsal horn neurons

Repeated peripheral noxious stimulation can induce long-lasting changes in synaptic efficacy, including LTP of C-fiber-evoked potentials, that are thought to critically contribute to pathologic pain and central sensitization in the spinal dorsal horn [37,38,39]. High-frequency afferent activity, where presynaptic activation is likely to coincide with a partially depolarized postsynaptic membrane, is assumed to best fulfill conditions for increased synaptic efficacy and information storage in neuronal circuits [40]. In the current study, we were able to induce LTP of C-fiber-evoked potentials during hyperdopaminergic transmission by using a low-frequency conditioning stimulation protocol that otherwise consistently fails to induce potentiation in basal, untreated rats (Fig 1). These findings support that threshold for synaptic potentiation in the dorsal horn by repeated noxious input is actually dynamic and regulated by descending dopaminergic input arising from the diencephalon, insofar as dopaminergic innervation of the spinal dorsal horn is provided primarily if not exclusively by neurons originating in the hypothalamic A11 cell group [41,42,43,44]. In addition, our data extend previous evidence supporting that dopamine can modulate the disposition of CNS neurons to undergo plastic changes in synaptic efficacy. For example, tonic dopamine levels facilitate LTP formation in rat prefrontal cortex [45], and dopamine or dopamine reuptake inhibitors decrease the thresholds for synaptic potentiation in the basolateral amygdala [46], prefrontal cortex [7] or hippocampus [47]. A primary finding of the current study is that hyperdopaminergic neurotransmission lowers the threshold for synaptic potentiation of responses to noxious input, in such a manner that afferent low-frequency input becomes effective at triggering LTP. Previous studies have reported that dopamine concentrations are increased at the spinal cord level during sustained nociception following intraplantar injection of carrageenan [17]. Here, we induced a local state of hyperdopaminergic neurotransmission by controlled superfusion of the lumbar cord dorsum with dopamine reuptake inhibitor GBR 12935, which was circumscribed to the lumbosacral dorsal horn segments innervated by the sciatic nerve. Although we did not attempt to measure dopamine levels after GBR 12935 administration, LFS may have induced sufficient dopamine release in the dorsal horn, considering that D1LR antagonist SCH 23390 partially prevented LTP induced by conditioning LFS (Fig 1). Furthermore, the blocking effect of SCH 23390 supports that dopaminergic modulation was at least in part mediated by D1LRs.

Whether increased dopamine concentration in the dorsal horn milieu is a common occurrence in the diverse forms of sustained or chronic pain remains to be established. Increasing evidence supports that dopaminergic input to the dorsal horn is indeed instrumental in descending modulation of pain via D1- and D2-type receptors, including the so-called diffuse noxious inhibitory controls (DNIC) [11]. However, our current findings predict that increased activity of dopaminergic mechanisms in the dorsal horn may at the same time result in decreased threshold to activity-dependent synaptic potentiation and ultimately higher vulnerability to mild peripheral input. Consistent with this view is previous evidence that strong, sustained pharmacological D1LR stimulation leads to late-onset potentiation of C-fiber-evoked potentials even in the absence of conditioning stimulation [48].

LTP failed to occur in the current study during superfusion with GBR 12935 in the absence of conditioning stimulation, indicating that hyperdopaminergic transmission does not suffice to generate LTP within the time-window evaluated here, but requires concurrent primary afferent input. Moreover, we found that LFS-induced LTP was dependent on activation of the NMDA receptor subunit 2B, as indicated by the blocking effect of NR2B antagonist Ro 25–6981 (Fig 1). This is in line with previous data supporting that afferent-induced LTP in dorsal horn neurons may depend on activation of the NMDA receptor [37,49,50], and specifically on NR2B [51,52]. Glutamate-dependent plasticity associated to spinal hyperexcitability in persistent pain states [53,54] involves enhanced activation of the NMDA receptor [29,13], presumably due to receptor permeation [55] or phosphorylation of specific subunits [56,57]. Switching the NMDA receptor to a high activation state in dorsal horn neurons is commonly assumed to be achieved by intense noxious input or conditioning high-frequency afferent stimulation [37,49,58,59,60,61]. In basal conditions, low-frequency stimulation in vivo either only inconsistently leads to LTP [39] or fails to do so as found here. Our current findings provide evidence of an alternative mechanism of switching the NMDA receptor to a higher activation state in dorsal horn neurons by mild afferent activity and concurrent increasing dopaminergic, D1LR-mediated input. Previous reports have shown that simultaneous D1L- and NMDA receptor-activation can enhance neural responses mediated by glutamatergic transmission elsewhere in the CNS [62,63,64]. Specifically, LTP induction in CA1 requires synergistic interaction between both these receptors [65], and dopamine priming in prefrontal cortical slices facilitates LTP by a mechanism involving coincidental NMDA receptor-activation [66,67]. Extensive interactions between D1L- and NMDA receptors have been reported that at least may involve heteromeric receptor complex formation and D1 receptor-mediated NMDAR phosphorylation [68]. At the dorsal horn level, recent evidence shows that spinal nerve ligation is followed by rapid, D1LR-dependent NMDA receptor phosphorylation and switching to a high activation state within 90 min after injury [16].

### Up-regulation of pDARPP-32^Thr34^ at postsynaptic, pNR2B^Tyr1472^-expressing neurons via convergent D1L- and NR2B receptor activation

Activation of DARPP-32 is involved in synaptic plasticity in the CNS [69,70]. In striatal neurons, DARPP-32 modulates NMDA receptors via PKA [62]. PKA promotes phosphorylation of threonine residues of DARPP-32 to facilitate synaptic plasticity [71,72], and depletion of PKA/DARPP32 signaling disrupts D1LR-related LTP in striatum [71]. We found that LTP of C-fiber-evoked potentials in the spinal dorsal horn, which involves concurrent NR2B/D1LR activation, is accompanied by changes in pDARPP-32 phosphorylation at Thr 34 in dorsal horn synapses 90 min after afferent LFS (Fig 2). Conditioning stimulation failed to increase pDARPP-32^Thr34^ levels *per se*, i.e. in the absence of a GBR 12935-induced hyperdopaminergic state, supporting that DARPP-32 phosphorylation was critically dependent on increased dopaminergic activity. Furthermore, we found that concurrent recruitment of NR2B was required for DARPP-32 phosphorylation to increase, as indicated by the strong attenuating effect of Ro 25–6981 (Fig 2). This result underscores that both LTP induction and pDARPP-32 phosphorylation are based on the premise of concurrent recruitment of D1L- and NMDA receptors. There is previous evidence that glutamate can trigger pDARPP-32 phosphorylation at threonine 34 in neostriatal neurons, probably via nNOS/NO/soluble guanylyl cyclase/cGMP/PKG signaling [73]. In order to evaluate whether the observed increase in pDARPP-32^Thr34^occurred in NMDAR-expressing neurons, and more specifically in dorsal horn neurons where NR2B is up-regulated after conditioning LFS, we quantitatively assessed co-localization of pDARPP-32^Thr34^with the phosphorylated form of 2B subunit on its Tyr1472 residue. In confirmation of the Western blot findings above, confocal immunofluorescence data showed that pNR2B^Tyr1472^ was dramatically up-regulated at postsynaptic sites in the dorsal during hyperdopaminergic transmission horn 90 min after LFS, as assessed by increased co-localization with PSD-95, a scaffolding protein present in postsynaptic densities (Fig 3). Further, we found that immunofluorescence for pDARPP-32^Thr34^ was highly co-localized with immunopositive signal for pNR2B^Tyr1472^ after LFS, confirming that pDARPP-32^Thr34^ largely occurs in neurons in which LFS induced functional up-regulation of NR2B. There is evidence implicating the NMDA subunit 2B is in spinal hyperexcitability ad pain, as NR2B is known to mediate allodynia and hyperalgesia during neuropathic pain [35,74] and increased phosphorylation of this protein on Tyr1472 has been reported in superficial laminae of dorsal horn after peripheral lesion [53,75]. Consistently, disruption of PSD-95 interaction with NR2B in dorsal horn neurons reduces pNR2B^Tyr1472^ and attenuates mechanical and thermal hypersensitivity in bone cancer pain [76]. Our current finding that pDARPP-32^Thr34^ and pNR2B^Tyr1472^ both are up-regulated and co-clustered at postsynaptic densities is highly suggestive of the involvement of DARPP-32 in synaptic potentiation in dorsal horn neurons. This view is consistent with the reported involvement of pNR2B^Tyr1472^in NR2B-dependent hippocampus LTP induced by chronic visceral pain [77], as well as with the reported prevention of ischemia-associated LTP by genetic pNR2B^Tyr1472^depletion [78]. Importantly, postsynaptic up-regulation of pDARPP-32^Thr34^ and pNR2B^Tyr1472^ that followed conditioning LFS was critically dependent on dopaminergic D1LR-mediated input, in light that it could be induced only during inhibition of dopamine re-uptake with GBR 12935 and was sensitive to D1LR blockade. These observations are in general agreement with previous reports showing that D1LR activation can both increase pNR2B^Tyr1472^ trafficking in prefrontal cortical neurons [33] and induce phosphorylation of carboxy-terminal terminus of NR2B in dendrite spines [34].

The current results support that convergence of glutamatergic and dopaminergic inputs to the spinal dorsal horn plays a critical role in regulating afferent-induced synaptic plasticity. Convergence of D1L- and NMDA receptor-mediated signals has been reported to modulate LTP in CA1 [79], and close interplay among DA receptors, DARPP-32 activity and glutamatergic transmission has been suggested to underlie cognitive aspects of striate function [69]. NMDAR/D1LR interaction appears to require postsynaptic mobilization of intracellular Ca+2 and protein kinase A (PKA) [64]. At the spinal cord level, PKA has been implicated in central sensitization and persistent pain [35,74]. Unfortunately, the current unavailability of drugs to selectively inhibit DARPP-32 precludes the possibility of directly assessing its participation in LTP formation. However, indirect evidence strongly supports this possibility. Experiments in KO mice for DARPP-32 have revealed that a D1LR-PKA-DARPP-32 pathway is critically involved both in corticostriatal LTP and LTD [69], and phosphorylation of DARPP-32 has been reported during LTP in the hippocampus-prefrontal pathway [70], where it has been interpreted as linked to gene expression for LTP maintenance. In addition, we show that convergent NR2B- and D1LR-mediated input is required both for inducing LTP of C-fiber-evoked evoked potentials and for postsynaptic pDARPP-32^Thr34^/pNR2B^Tyr1472^ up-regulation in dorsal horn neurons. By virtue of its unique position to act as detection sensor of convergent glutamate and dopamine inputs to the dorsal horn, DARPP-32 may play a role in spinal hyperexcitability and pain by regulating the threshold for afferent-induced synaptic potentiation.
